# Detection of suicidality from medical text using privacy-preserving large language models

**DOI:** 10.1192/bjp.2024.134

**Published:** 2024-12

**Authors:** Isabella Catharina Wiest, Falk Gerrik Verhees, Dyke Ferber, Jiefu Zhu, Michael Bauer, Ute Lewitzka, Andrea Pfennig, Pavol Mikolas, Jakob Nikolas Kather

**Affiliations:** Else Kroener Fresenius Center for Digital Health, Technical University Dresden, Dresden, Germany; and Department of Medicine II, Medical Faculty Mannheim, Heidelberg University, Mannheim, Germany; Department of Psychiatry and Psychotherapy, Carl Gustav Carus University Hospital, Technical University Dresden, Dresden, Germany; Else Kroener Fresenius Center for Digital Health, Technical University Dresden, Dresden, Germany; National Center for Tumor Diseases (NCT), Heidelberg University Hospital, Heidelberg, Germany; and Department of Medical Oncology, Heidelberg University Hospital, Heidelberg, Germany; Else Kroener Fresenius Center for Digital Health, Technical University Dresden, Dresden, Germany; Else Kroener Fresenius Center for Digital Health, Technical University Dresden, Dresden, Germany; National Center for Tumor Diseases (NCT), Heidelberg University Hospital, Heidelberg, Germany; Department of Medical Oncology, Heidelberg University Hospital, Heidelberg, Germany; and Department of Medicine I, University Hospital Dresden, Dresden, Germany

**Keywords:** Large language models, natural language processing, suicidality, psychiatric disorder detection, electronic health records

## Abstract

**Background:**

Attempts to use artificial intelligence (AI) in psychiatric disorders show moderate success, highlighting the potential of incorporating information from clinical assessments to improve the models. This study focuses on using large language models (LLMs) to detect suicide risk from medical text in psychiatric care.

**Aims:**

To extract information about suicidality status from the admission notes in electronic health records (EHRs) using privacy-sensitive, locally hosted LLMs, specifically evaluating the efficacy of Llama-2 models.

**Method:**

We compared the performance of several variants of the open source LLM Llama-2 in extracting suicidality status from 100 psychiatric reports against a ground truth defined by human experts, assessing accuracy, sensitivity, specificity and F1 score across different prompting strategies.

**Results:**

A German fine-tuned Llama-2 model showed the highest accuracy (87.5%), sensitivity (83.0%) and specificity (91.8%) in identifying suicidality, with significant improvements in sensitivity and specificity across various prompt designs.

**Conclusions:**

The study demonstrates the capability of LLMs, particularly Llama-2, in accurately extracting information on suicidality from psychiatric records while preserving data privacy. This suggests their application in surveillance systems for psychiatric emergencies and improving the clinical management of suicidality by improving systematic quality control and research.

Attempts to apply artificial intelligence (AI) and machine learning to detection of psychiatric disorder have yielded only moderate accuracy owing to small effect sizes and high heterogeneity.^1^ Nevertheless, improving prediction models by incorporating clinical assessments seems to enable clinical applications.^2^ However, a significant challenge arises from the nature of clinical data: medical free text, especially in psychiatry, encapsulates a wealth of information about a patient's pathology and well-being by unveiling the structure of their thinking and feeling. This information is vital but often remains inaccessible for scalable analysis because of its unstructured nature. The inability to effectively analyse this text on a large scale potentially leads to missed opportunities in clinical decision-making and research.

Recent studies have emphasised the significant impact of advanced technology on managing unstructured medical data.^3^ Specifically, the use of large language models (LLMs) has garnered significant attention.^4^ Unlike previously used methods of natural language processing (NLP) that require decomposing the text and substantial feature engineering,^5^ LLMs are AI models primarily designed to understand and generate text.^6^ They are trained on vast amounts of text data, allowing them to learn the statistical patterns and relationships within language.^7^

Accounting for nearly half of all emergency psychiatric admissions,^8^ suicide is one of the most tragic complications of psychiatric care and is often preventable. Sustained efforts can lead to major reductions in in-patient suicides, from 4.2 to 0.74 per 100 000 admissions.^9^ Here, we hypothesise that automated tools could help identify in-patient suicide risk using underexploited clinical records. Moreover, beyond clinical application, LLMs might automatically identify and extract suicidality from electronic health records (EHRs) to enhance research.

## Method

**Fig. 1:**
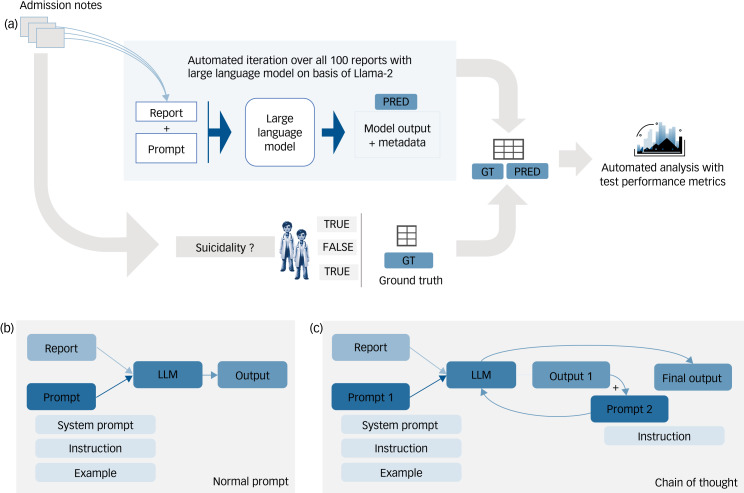
Experimental Setup. (a) The information extraction pipeline. The psychiatry reports (*n* = 100) were transferred to a csv table. Our pipeline then iterates over all reports with the predefined prompt and outputs a JavaScript Object Notation-File (JSON) file with all Large Language Model (LLM) outputs (PRED). The relevant classes (suicidality present: yes or no) were then extracted from the LLM output, which was more verbose in some cases. These outputs were then transferred to a pandas dataframe and automatically compared to the expert-based ground truth (GT). (b) The initial prompting strategy. One prompt and one report were given to the model at the same time. Every prompt contained a system prompt with general instructions and a specific question to the report (Instruction). (c) The chain-of-thought approach: the psychiatry report with our prompt was fed into the LLM, which generated a first output. With a second prompt and a predefined answering grammar, the model was fed its own output and again forced to generate a certain, json based output structure. This final output then underwent performance analysis. Icon Source: Midjourney.

We systematically extracted *n* = 100 randomly selected text-based admission notes of in-patients treated in and discharged from the acute psychiatric ward of the Department of Psychiatry and Psychotherapy at the University Hospital Carl Gustav Carus Dresden between 1 January and 31 December 2023. A typical, though fictitious account (to preserve privacy) can be found in the Supplementary material available at https://doi.org/10.1192/bjp.2024.134. We included 54 female and 46 male patients with an average age of 50 years (range 18–96 years, s.d. = 23.8 years). The most prevalent ICD-10 main diagnoses were major depressive disorder (21%), psychotic disorders (20%) and dementia (17%) (Table 1). Suicidality evaluation is part of the unedited input data, as assessment is a required care standard.^10^ However, this assessment is generally not documented in our EHRs in a structured way. Instead, the rater describes their impression, for example stating that no suicidal ideation was apparent. Variations in expressing this assessment (sometimes without mentioning ‘suicidal intent’ at all) and negations are common (e.g. ‘(no) reason to assume suicidal ideation’, ‘suicidal intent (not) clearly ruled out’, ‘wish to be dead present’), which reduced efficiency in earlier NLP assessments.^11^ We ensured data privacy by installing Llama-2 via the llama.cpp framework on a local hospital computer. We extracted the suicidality status from psychiatric admission notes using three different Llama-2-based models: the standard English Llama-2-70b chat model adapted to allow deployment on low-resource consumer hardware,^12^ as well as two versions of Llama-2 that were specifically fine-tuned for the German language (‘Sauerkraut’^13^ and ‘Emgerman’^14^). We compared the models’ results with a ground truth consensus which was established by a resident (F.G.V.) and a consultant psychiatrist (P.M.) as a binary variable (suicidal/not suicidal) (Fig. 1). Suicidality was defined as either suicidal thoughts, ideation, plans or attempt identified by hospital admission. We applied a step-by-step approach to prompt engineering, as prompt engineering can substantially improve the performance of LLMs.^15^ The first prompt simply asked about suicidality in reports (P0). In the second prompt, we added fictitious examples and explanations. We started with one example (P1) and added one example (P2) at a time, with three examples as a maximum (P3) (Supplementary Table 1). After achieving improved performance, we incorporated a chain-of-thought approach (Fig. 1(c)). For this, the model was prompted to identify whether a patient exhibited suicidal thoughts and to provide an explanation based on the given input. Subsequently, the model's output – specifically its reasoning about suicidality – was used as the basis for a second prompt. In this subsequent interaction, the model was tasked with providing a binary response (true or false) regarding the presence of suicidality (P4). To obtain reliable estimates, we used bootstrapping, a statistical resampling technique, with 10 000 iterations.

**Table 1:** Patient characteristics (*n* = 100)

Characteristic	%
Gender	
Male	46
Female	54
Age, years	
18–24	18
25–44	31
45–64	24
65–84	12
85+	15
Main diagnosis	
Major depressive disorder	21
Psychotic disorders	20
Dementia	17
Borderline personality disorder	9
Alcohol use disorder	8
Schizoaffective disorders	8
Other	17

All research procedures were conducted in accordance with the Declaration of Helsinki. Ethics approval was granted by the ethics committee of Technical University Dresden (reference number BO-EK-400092023). Informed consent was not necessary for this study because the research involved data from which all personal identifiers had previously been removed. The design of the study ensured that there was no interaction or intervention with participants and no potential for harm or invasion of privacy.

## Results

**Fig. 2:**
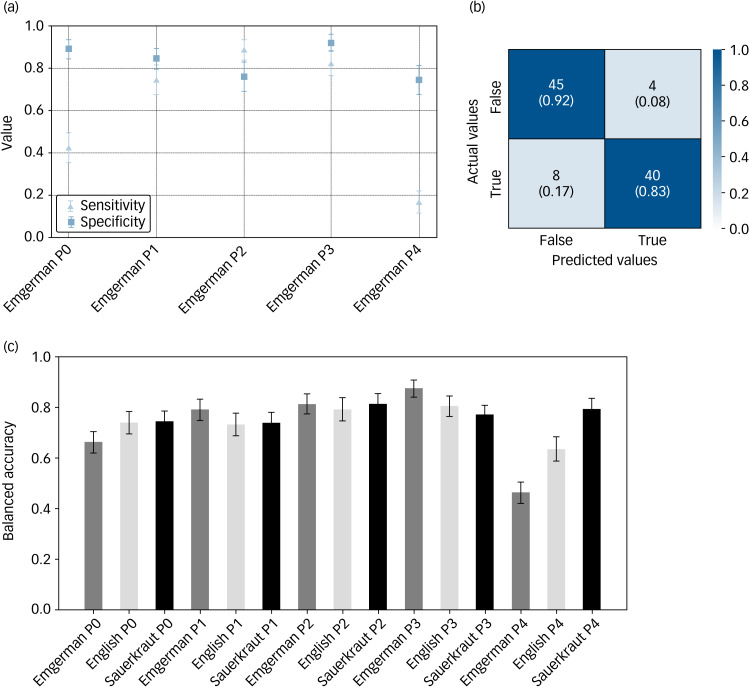
Performance of German-language fine-tuned Llama-2 model. (a) Sensitivity and Specificity for five different prompting strategies. With P0, the model was simply asked to provide the answer if suicidality was present from the report, P1, P2 and P3 provided one, two or three examples to the model. P4 applied a chain-of-thought approach, where the model was asked twice, with the first model output as input for the second run. (b) Confusion matrix representing the performance of the Large Language Model (LLM) indicating the presence of suicidality based on the examined admission notes (*n* = 100) with a sensitivity of 83% as well as specificity of 92% for P3, a prompt that included three examples. (c) Bar chart showing the balanced accuracies for all models and prompt engineering attempts. Error bars show the 95% confidence interval of the bootstrapped samples.

Llama-2 extracted suicidality status from psychiatric reports with high accuracy across all five prompt designs and all three models tested. The highest overall accuracy was achieved by one of the German fine-tuned Llama-2 models (‘Emgerman’), which correctly identified suicidality status in 87.5% of the reports. With a sensitivity of 83.0% and a specificity of 91.8%, it demonstrated the highest balanced accuracy of all models (87.4%) (Fig. 2(a)).

The confusion matrix (Fig. 2(b)) also highlights areas for model improvement, particularly in reducing false negatives. To improve the performance, we designed the prompts and developed five different prompting strategies that were tested for all three models (Fig. 2(c)). The simplest prompt, which contained only a ‘system prompt’ framing the model in its role (‘You are an attentive medical assistant with specialised knowledge in psychiatry [ … ]’) one report at a time and the ultimate question of interest (‘Is the patient suicidal? Answer yes or no [ … ]’), yielded the highest sensitivity in the German fine-tuned Llama-2 model ‘Sauerkraut’ (sensitivity: 87.5%, specificity: 61.2%, balanced accuracy: 74.4%). It was immediately followed by the standard English Llama-2 chat model, with a sensitivity of 85.1%, specificity of 63.0% and a balanced accuracy of 74.1%. The Emgerman model had a worse sensitivity (42.6%), but the highest specificity (98.8%). Not all models improved when examples were added to the prompt, allowing for in-context learning. The Emgerman model improved substantially by adding more examples, with the lowest balanced accuracy in the prompt with no examples given (66.2%) and the highest balanced accuracy in the prompt with three examples given (87.4%). The English model was robust, showing similar balanced accuracies for prompts with no, one, two or three examples (P0: 74.1%; P1: 73.3%; P2: 79.3%; P3: 80.3%). The ‘Sauerkraut’ model improved with adding examples but achieved its maximum performance with two examples in the prompt. The use of the chain-of-thought approach did not improve performance (sensitivities: ‘Emgerman’ P4 17.0%, ‘English’ P4 63.8%, ‘Sauerkraut’ P4 81%; specificities: ‘Emgerman’ P4 75.5%, ‘English’ P4 63.3%, ‘Sauerkraut’ P4 77.6% (Table 2)). In fact, all models deteriorated, except for the ‘Sauerkraut’ model, which was not affected negatively by this approach.

**Table 2:** Performance metrics of the three tested large language models (‘Emgerman’, ‘Sauerkraut’, ‘English’) with the five prompt variations (P0–P4)^a^

Model	Accuracy		PPV		Sensitivity		Specificity		NPV		F1 score		Balanced accuracy	
	Mean	s.d.	Mean	s.d.	Mean	s.d.	Mean	s.d.	Mean	s.d.	Mean	s.d.	Mean	s.d.
Emgerman P0	0.667	0.048	0.8	0.082	0.426	0.073	0.898	0.044	0.62	0.057	0.552	0.072	0.662	0.042
Emgerman P1	0.793	0.041	0.815	0.059	0.746	0.064	0.837	0.053	0.775	0.058	0.777	0.049	0.792	0.041
Emgerman P2	0.812	0.04	0.773	0.058	0.872	0.049	0.754	0.062	0.86	0.053	0.818	0.042	0.813	0.039
Emgerman P3	0.875	0.034	0.907	0.044	0.83	0.055	0.918	0.039	0.849	0.049	0.865	0.039	0.874	0.034
Emgerman P4	0.468	0.051	0.4	0.112	0.17	0.055	0.755	0.062	0.486	0.057	0.236	0.069	0.463	0.041
English P0	0.741	0.046	0.7	0.061	0.851	0.052	0.629	0.072	0.805	0.067	0.767	0.047	0.74	0.045
English P1	0.731	0.045	0.703	0.062	0.792	0.059	0.672	0.067	0.767	0.066	0.743	0.049	0.732	0.045
English P2	0.788	0.048	0.731	0.069	0.881	0.055	0.703	0.074	0.866	0.062	0.797	0.052	0.792	0.046
English P3	0.805	0.04	0.854	0.055	0.73	0.065	0.878	0.047	0.768	0.057	0.785	0.048	0.804	0.04
English P4	0.635	0.049	0.625	0.07	0.638	0.071	0.633	0.068	0.646	0.069	0.629	0.058	0.636	0.049
Sauerkraut P0	0.742	0.044	0.689	0.059	0.875	0.048	0.612	0.07	0.833	0.063	0.769	0.045	0.743	0.042
Sauerkraut P1	0.742	0.044	0.897	0.057	0.542	0.072	0.939	0.034	0.677	0.056	0.672	0.062	0.74	0.04
Sauerkraut P2	0.815	0.039	0.858	0.054	0.749	0.062	0.878	0.047	0.781	0.056	0.798	0.047	0.814	0.039
Sauerkraut P3	0.773	0.042	0.964	0.035	0.562	0.071	0.98	0.02	0.696	0.055	0.708	0.06	0.771	0.037
Sauerkraut P4	0.793	0.042	0.777	0.059	0.81	0.057	0.776	0.06	0.81	0.057	0.791	0.046	0.793	0.042

PPV, positive predictive value; NPV, negative predictive value.

a. All results were obtained by 10 000-fold bootstrapping, and therefore means and standard deviations are given.

## Discussion

We show that large language models (LLMs) demonstrate remarkable efficacy in identifying and extracting references to suicidality from psychiatric reports. Their performance, in terms of both sensitivity and specificity, was notable and improved progressively with the number of examples provided in the prompt. These findings suggest a significant advancement in the field, highlighting the potential of LLMs to revolutionise the way psychiatric medical text is analysed. In contrast to traditional natural language processing (NLP) methods, which require extensive annotation or model training, our approach uses the capabilities of the foundation models’ inference and is applicable to comparatively small data-sets. The real-life clinical data taken from an acute care ward in a maximum care facility in a German urban centre was processed at the ‘edge’ – with no need to upload to commercial servers or a data-processing cloud – by an open-source model on local servers. This enables a privacy-sensitive data protection strategy in a closed loop, that alleviates concerns about data leaving the care provider's control.

The good performance levels (Fig. 2) even in a (medical) domain in which the LLM was not fine-tuned suggest even greater opportunities with further optimisation for mental health, for example in dealing with physician-level linguistic idiosyncrasies or abbreviations.^16^ For a clinical application such as suicide risk detection, where false negatives are likely to lead to detrimental outcomes, sensitivity should approach 100%, even at the cost of detecting more false positives, which can be resolved with further human evaluation to ensure no case is missed. In any clinical setting, the final risk assessment remains in the judgement of the experienced clinician and further research needs to elucidate risks and challenges. On the other hand, in the case of data extraction for research purposes, correctly identifying 80% of cases (i.e. classification accuracy of 80%) might be adequate to capture a representative cohort. In comparison, randomised clinical trials for major depression may include less than a quarter of cases from real-life clinical cohorts, owing to strict eligibility criteria.^17^

Other clinical applications could include prediction and early warning of deterioration in symptom severity and a subsequent need for escalation of therapy, such as involuntary admission, restraint or forcible medication. Multiprofessional communication in interdisciplinary care provider teams including nurses, specialty therapists, psychotherapists and psychiatrists might also become more efficient, for example owing to reduced information loss during handover or case conferences.

### Strengths and limitations

Our approach allows an out-of-the box application, whereas classic NLP approaches require time-consuming training and data annotation^20^ and present limited^21,22^ or comparable performance.^23^ In addition, the performance of the basic language models trained on large corpora of a variety of text data not specific to our data-set suggests good generalisability.^18^ The comparatively small need for computational resources, since they are used only for inference, not for specific training,^19^ allows for easy application at the point of origin of the data and may therefore be more scalable than classic NLP approaches.

The potential generalisability of our approach is supported by the fact that many physicians were involved in the creation of the clinical letters and it is highly unlikely that the notes used reflect a personal style of any particular resident. On average, 50% of the patients on our acute ward were admitted during the night shifts. On average, 20 residents rotate through the night shifts on a daily basis. Acute ward residents rotate on a 3- to 6-month basis. However, we acknowledge that a clinic-specific style may play a role. In the next step, reproducibility should be tested on a larger external validation sample.

Suicide risk was considered a binary parameter. Future research should concentrate on a more detailed outcome that differentiates between overall suicide risk and acute high risk.^24^ Additionally, studies should apply extensive ground truth labelling,^25^ and evaluate more comprehensive prompt engineering strategies.^26^ However, our results suggest that, at least in the case of Llama-2, more complex prompting with a chain-of-thought approach might degrade performance. For some tasks, simple example prompting that requires very few computing resources may be more suitable.

Although patient privacy concerns have been addressed, it is important to note that every LLM approach inherits ethical issues related to bias, trust, authorship and equitability.^27^ Expert guidelines for development of LLMs for medical purposes should be carefully considered.^28^

### Conclusions

We provide a proof-of-concept analysis for automated extraction of references to suicidality in in-patients from EHRs using LLMs. This study highlights the transformative potential of using LLMs to detect suicidality in clinical admission notes. The use of a psychiatry-naive model, not specifically fine-tuned to the relevant data-sets, shows high performance, which is promising for generalisability and offers potential for further improvement through more extensive in-context learning and prompt engineering. Possible applications include early warning and surveillance tools for psychiatric emergencies, preventing information transfer failures, quality assurance and evaluation of psychiatric symptoms on large clinical ‘real-world’ samples.

## Supporting information

Wiest et al. supplementary material Wiest et al. supplementary material

## Data Availability

The data used in this study are not available for sharing because they contain information that could compromise the privacy of the research participants. The source code necessary for replicating our procedures are openly available to other researchers at https://github.com/I2C9W/LLM4Psych/tree/v0.1.0.
