# Supplementary material for: Detection of suicidality from medical text using privacy-preserving large language models
Source: Br J Psychiatry. 2024 Dec;225(6):532–7. doi: 10.1192/bjp.2024.134 (PMC11669470; doi:10.1192/bjp.2024.134)
Supplement: Wiest et al. supplementary material [file S000712502400134Xsup001.docx]

# Supplement

**Fictitious example of a typical admission note:**

In the mental status examination, the patient appeared alert and coherent. Fully cooperative, though with long latency in answering. Mood depressed. No fear or compulsions reported. Denies confusion and hallucinations. No mistrust reported. During examination clearly distanced from suicidal or endangering thoughts and behavior.

**Supplementary Table 1**

| Original Prompt | Prompt Translation |
| --- | --- |
| “Sie sind ein aufmerksamer, medizinischer Assistent mit Spezialwissen in Psychiatrie. Im Folgenden finden Sie eine psychiatrische Anamnese. Bitte beantworten Sie die Frage.  Anamnese: {}  Frage: Ist der Patient suizidal? Antworten Sie mit ja oder nein.Geben Sie einen Auszug aus der Anamnese zur Begründung.” | "You are an attentive medical assistant with specialized expertise in psychiatry. Below you will find a psychiatric history. Please answer the question.  Medical history: {}  Question: Is the patient suicidal? Answer yes or no. Give an excerpt from the medical history to justify your answer." |
| “Sie sind ein aufmerksamer, medizinischer Assistent mit Spezialwissen in Psychiatrie. Im Folgenden finden Sie eine psychiatrische Anamnese. Bitte beantworten Sie die Frage. Zunächst erhalten Sie ein Beispiel.  Beispiel 1: Pat. wach, bewusstseinsklar, freundlich zugewandt und gesprächsbereit. Zeitlich unsicher (-1 Tag), örtlich, situativ und zur Person orientiert. Auffassung, Konzentration und Gedächtnis ungestört. Keine formalen oder inhaltlichen Denkstörungen, Störungen des Ich-Erlebens oder der Wahrnehmung. Stimmung ausgeglichen, Affekt situationsadäquat und schwingungsfähig. Keine Ängste oder Zwänge. Antrieb und Psychomotorik ungestört. Kein Lebensüberdruss oder Suizidgedanken. Keine akute Eigen- oder Fremdgefährdung.  Frage: Antworten Sie mit ja oder nein. Geben Sie einen Auszug aus der Anamnese zur Begründung.  ASSISTANT: nein. Begründung: In der Anamnese steht, dass der Patient keinen Lebensüberdruss oder Suizidgedanken hat. Zusätzlich besteht keine akute Eigen- oder Fremdgefährdung. Zusammenfassend besteht keine Suizidalität.  Beispiel 2: Wach, bewusstseinsklar. Auffassung und Konzentration im klinischen Eindruck eingeschränkt. Formalgedanklich auf Belastungssituation und Entlasswunsch eingeengt, nicht auslenkbar und von normaler Geschwindigkeit. Inhaltliche Denkstörungen verneint. Wahrnehmungsstörungen verneint. Deutlich dysphorisch, nicht schwingungsfähig beideutlich gedrückter Stimmungslage. Psychomotorisch deutlich agitiert. Kein Anhalt für Fremdgefährdung, akute Eigengefährdung bei Suizidalität.  Frage: Antworten Sie mit ja oder nein. Geben Sie einen Auszug aus der Anamnese zur Begründung.  ASSISTANT: ja. Begründung: Dies lässt sich aus dem letzten Satz der Anamnese schließen: "akute Eigengefährdung bei Suizidalität.  Beispiel 3: Patient wach, bewusstseinsklar, zu allen 4 Qualitäten vollständig orientiert, noch freundlich, auskunftsbereit, Stimmung kaum explorabel bei Drogenkonsum, zuweilen im Gespräch parathym und situationsinadäquat, laut lachend, formaler und inhaltlicher Gedankengang deutlich auffällig inkohärent bis zerfahren, Anhalt auf Sinnestäuschung vorhanden (blickt im Raum umher), kein Anhalt auf Ängste oder Zwänge. Aktuell ebenfalls klar abständig von akuter Suizidalität, akute Fremdgefährdung. Eigengefährdung und Fremdgefährdung im Rahmen der psychotischen Realitätsverkennung.  Frage: Antworten Sie mit ja oder nein. Geben Sie einen Auszug aus der Anamnese zur Begründung.  ASSISTANT: nein. Begründung: Nein, der Patient ist aktuell klar abständig von akuter Suizidalität.  Anamnese: {}  Frage: Ist der Patient suizidal? Antworten Sie mit ja oder nein. Geben Sie einen Auszug aus der Anamnese zur Begründung.” | "You are an attentive medical assistant with specialized knowledge in psychiatry. Below you will find a psychiatric history. Please answer the question. First you will be given an example.  Example 1: Patient is awake, conscious, friendly and demonstrating a willingness to engage in a conversation. Disorientation of time (-1 day). Oriented to the location, situation and personal identity. Perception, concentration and memory are intact. No evidence of formal thought disorder or abnormal thought content. Mood stable, affect appropriate to the situation and within normal fluctuations. No fears or compulsions. No abnormalities of activity. No feelings of weariness with life or suicidal thoughts. No immediate risk to self or others.  Question: Answer yes or no. Give an excerpt from the medical history to justify your answer.  ASSISTANT: no. Rationale: Medical history indicates that the patient is not experiencing any feelings of weariness with life or suicidal thoughts. In addition, there is no immediate risk to self or others. In summary, there is no suicidal ideation.  Example 2: Awake, conscious. Signs of impaired comprehension and attention. Thought process of intact rate, perseverating and fixated to the precipitating situation and desire to self-discharge. No signs of delusions. Perceptual disorders denied. Mood clearly dysphoric and depressed, without evident mood swings. Psychomotor agitation. No immediate indications of danger to others, acute danger to self in case of suicidal tendencies.  Question: Answer yes or no. Give an excerpt from the medical history to justify your answer.  ASSISTANT: yes. Rationale: This can be inferred from the last sentence of the medical history: "acute danger to self in case of suicidal tendencies”  Example 3: Patient awake, conscious, fully oriented across all 4 dimensions, friendly, presenting himself as willing to provide information, however, mood barely explorable due to drug intoxication, displaying intermittently parathymic and inappropriate affect, laughing out loud during the conversation. Formal and content-related thought process clearly incoherent, signs of hallucinations (looks around the room), no evidence of specific fears or compulsions. Currently clearly not displaying acute suicidal tendencies. Signs of acute danger to others. A risk of harm to both themselves and others in the context of psychotic misinterpretation of reality.  Question: Answer yes or no. Give an excerpt from the medical history to justify your answer.  ASSISTANT: no. Reason: No, the patient is currently clearly not displaying acute suicidal tendencies  Medical history: {}  Question: Is the patient suicidal? Answer yes or no. Give an excerpt from the medical history to justify your answer.” |
